# Investigating Low‐Temperature Stress Responses in Crustacea Aquatic Species Through Comparative Transcriptomics

**DOI:** 10.1111/eva.70254

**Published:** 2026-05-12

**Authors:** Ying Chen, Weihua Yan, Tong Xu, Shengyu Xu, Qifan Zeng, Zhe Qu, Zhenmin Bao, Hao Wang

**Affiliations:** ^1^ MOE Key Laboratory of Marine Genetics and Breeding, College of Marine Life Sciences/Key Laboratory of Tropical Aquatic Germplasm of Hainan Province Sanya Oceanographic Institution, Ocean University of China Qingdao Shandong/Sanya, Hainan China; ^2^ Hainan Lanyin Aquatic Seed Industry Technology Co., Ltd Wenchang Hainan China; ^3^ Southern Marine Science and Engineering Guangdong Laboratory (Guangzhou) Guangzhou China; ^4^ Hainan Seed Industry Laboratory Sanya Hainan China

**Keywords:** comparative transcriptome, crustaceans, low‐temperature stress, orthogroups

## Abstract

Crustaceans, such as shrimp and crabs, are pivotal to global aquaculture, yet their productivity is severely impacted by low‐temperature stress. This study employs comparative transcriptomic and genomic analyses to elucidate the molecular mechanisms underlying crustacean responses to cold stress across five economically significant species: *Litopenaeus vannamei* , *Penaeus indicus* , *Eriocheir sinensis* , *Cherax quadricarinatus* , and *Macrobrachium rosenbergii* . We identified 4711 conserved orthogroups and analyzed differential gene expression under low‐temperature conditions. Results revealed species‐specific responses, with 25 orthogroups shared across all five species and 113 orthogroups common to at least four species. Functional enrichment highlighted pathways such as oxidation–reduction, hormone metabolism, immune regulation, and spliceosomes. Key genes, including *EcKL*, *CYP2W1*, *HSPA5*, and *SLCs*, were implicated in signaling, metabolic, immunological, and developmental adaptations. Phylogenetic analysis was used to trace the evolutionary origins of stress‐responsive genes, enabling the identification of both ancient conserved genes and lineage‐specific genes. The results indicated that “younger” genes tend to exhibit greater transcriptional plasticity than ancient conserved genes. These findings provide critical insights into shared and species‐specific low‐temperature adaptation mechanisms, offering a foundation for breeding cold‐tolerant crustacea strains to enhance aquaculture resilience.

## 1 Introduction

Crustaceans, including shrimp and crabs, etc., play a crucial role in the global aquaculture industry due to their high economic value and substantial market demand, particularly in food consumption and export trade (Verdegem et al. 2023). As the largest producer of crustaceans worldwide, China significantly influences both global production volume and market dynamics. In 2022, China's crustacean aquaculture production surpassed 7.0 million tons, accounting for nearly 40% of total global output, while marine aquaculture primarily concentrated on shrimp and crabs (FAO 2024). Numerous factors influence aquaculture yields, with temperature regulating and constraining all physiological and behavioral parameters of ectothermic organisms (Abram et al. 2017).

Crustaceans exhibit a high sensitivity to variations in water temperature. While low‐temperature conditions below 20°C are commonly utilized during the commercial transportation of shrimp and crabs to lower their metabolic rates and ensure survival, low temperatures during the cultivation process can adversely affect production, ultimately restricting the growth of the aquaculture industry (Lagerspetz and Vainio 2006). For instance, the optimal growth temperature for *Litopenaeus vannamei* (Boone, 1931) ranges from 25 to 32 degrees Celsius. Below 18 degrees, feeding ceases, and below 9 degrees, mass mortality occurs (Ponce‐Palafox et al. 1997). Similarly, the suitable growth temperature for *Cherax quadricarinatus* (redclaw crayfish) is between 24 and 30 degrees Celsius; feeding also ceases at 14 degrees, resulting in death approximately 4 weeks later (Azra et al. 2020; Haubrock et al. 2021).

In crustaceans subjected to cold stress, metabolic rates lead to reduced feeding, stunted growth, and prolonged aquaculture cycles. Low temperatures compromise immune system functions, increasing susceptibility to diseases such as shell disease, while also potentially altering aquatic microbial communities and promoting the proliferation of pathogens (Wang and Chen 2006). The sex differentiation and gonad development of certain crustaceans are temperature‐dependent; low temperatures may negatively impact reproductive efficiency (e.g., low temperatures can promote feminization, whereas extreme low temperatures may cause abnormal gonad development) (Qiao et al. 2015). Although low temperatures increase dissolved oxygen levels in the water, the slowed metabolism can result in the accumulation of uneaten feed and excretion products, leading to elevated levels of harmful substances such as ammonia nitrogen (Zhang et al. 2009). However, as a Northern Hemisphere nation, China's marine aquaculture has long been affected by low temperatures, particularly in the northern regions during winter. Even in the southernmost provinces, such as Hainan and Guangdong, winter temperatures can drop to as low as 15°C, severely impacting the yields of Crustacea aquatic species. Thus, investigating the mechanisms of gene regulation in response to low‐temperature stress is crucial for breeding low‐temperature‐tolerant strains and for the aquaculture industry under winter low‐temperature conditions.

Currently, transcriptomics is extensively utilized as a conventional approach to elucidate the mechanisms underlying low‐temperature stress in crustaceans, with a substantial amount of relevant sequencing data available across various species. However, previous studies have predominantly concentrated on single‐species research, with comparatively less emphasis placed on interspecies comparisons (Lin et al. 2025; Mitalo et al. 2022; Wang et al. 2024). Actually, the combined analysis of comparative genomics and comparative transcriptomics can more effectively integrate evolutionary inquiries with mechanistic investigations (Breschi et al. 2017; Yao et al. 2022). Furthermore, analyzing the molecular mechanisms by which representative crustacean species respond to low‐temperature stress is essential for identifying both common and species‐specific key genes, which are critical for the targeted development of molecular tools aimed at mitigating low‐temperature stress damage.

Here, we performed comparative genomics and comparative transcriptomics analysis of crustaceans subjected to low‐temperature stress, identifying direct homologs of genomes across various evolutionary lineages. It also identified low‐temperature stress response genes and conducted functional and pathway analyses for five economically significant crustacean species. Based on their functions, the response stages were categorized, and the findings provide valuable data resources for elucidating the gene regulatory mechanisms associated with low‐temperature stress. This research contributes to a deeper understanding of how species adapt to low‐temperature environments throughout the evolutionary process.

## 2 Methods

### 2.1 Data Collection

For the comparative genomic analysis of crustaceans and the construction of a Metazoa species phylogenetic tree, we selected 70 species to identify orthologs, including 33 Ecdysozoa (comprising 24 Arthropoda, 6 Nematoda, 1 Priapulida, and 2 Tardigrada), 15 Lophotrochozoa, 19 Deuterostomia (including 1 Cephalochordata, 12 Craniata, 3 Echinodermata, 1 Hemichordata, and 2 Urochordata), and 3 non‐bilateral outgroup species (Cnidaria, Placozoa, and Porifera) (Table S1). This selection was utilized to build the data matrix. To analyze the gene expression regulatory mechanisms of low‐temperature stress in crustaceans, we collected low‐temperature stress transcriptomic data from five representative Crustacea species, along with comparable data from Drosophila. Detailed sample information, including temperature conditions, tissue types, and biological replicates, is provided in Table S1.

### 2.2 Homology Determination and Phylogenetic Analysis

Orthofinder software was utilized for all‐to‐all homology comparisons to generate a matrix of homologous genes across all species (Emms and Kelly 2019). Based on the evolutionary relationships among species, genes were classified according to their evolutionary lineages, employing the classification methods referenced from the study conducted by (Ma and Zheng 2023). 78 orthologs were selected from Orthogroups clustered to construct a species phylogenetic tree. These orthologs satisfied these conditions: (a) 100% taxon occupancy for each species; (b) ≥ 50% taxon are single‐copy genes. Mafft (version 7.221) was used to align amino acid sequences with options “‐‐thread 4 ‐‐auto ‐‐maxiterate 1000” (Katoh and Standley 2016). Gblocks was used to trim gene alignment with options “‐gappyout ‐col‐numbering” to obtain a conserved block (Castresana 2000). For all trimmed alignment, we inferred the concatenation‐based ML tree using IQ‐TREE (v1.6.12) with options “‐m TEST ‐seed 668688 ‐nt AUTO ‐bb 1000” with the LG + F + I + G4 optimal amino acid substitution model (Minh et al. 2020).

### 2.3 Transcriptome Profiling and Characterization

The genome assembly of six species were derived from NCBI genome database (Table S1). Salmon software was utilized for aligning transcript reads and quantifying gene expression (Noda et al. 2019). A total of 4711 orthologs were used to perform principal component analysis (PCA) among different species and experimental conditions, with the factoextra R package (Lê et al. 2008). Differential expression analysis was conducted using the DESeq2 R package (Love et al. 2014). Genes meeting the criteria of FDR < 0.05 and |log_2_FC| > 1.5 were considered significantly differentially expressed genes (DEGs), while those with FDR < 0.01 and |log_2_FC| > 2 were classified as highly significantly differentially expressed genes (HDEGs). Gene ontology (GO) and Kyoto Encyclopedia of Genes and Genomes (KEGG) enrichment analyses of DEGs were performed using the EnrichmentPipeline.

### 2.4 Gene Co‐Expression Network Analysis

The gene co‐expression network was constructed with the WGCNA R package (Langfelder and Horvath 2008). We employed the hypergeometric test to identify and enrich genes associated with the response to low‐temperature stress within the modules, with the objective of elucidating the modules that respond to low‐temperature stress in the co‐expression network. The Gephi software was utilized for visualizing the hub genes within the stress response modules (Bastian et al. 2009).

## 3 Result

### 3.1 Comparative Genomic Analysis of Crustacea Species

To investigate the shared and species‐specific genes related to the stress responses of Crustacea aquatic animals under low‐temperature conditions, we gathered whole‐genome data from 70 species, classified by their primary evolutionary lineage for comparative genomic analysis. This dataset includes 48 Protostomes, 19 Deuterostomes, and 3 basal taxa. Among the Protostomes, there are 15 Lophotrochozoans and 33 Ecdysozoans; the latter group comprises 24 Arthropods and 9 Nematodes as outgroups. Notably, within the Arthropods, 15 species are classified as Crustaceans and 9 as Insects.

**FIGURE 1:**
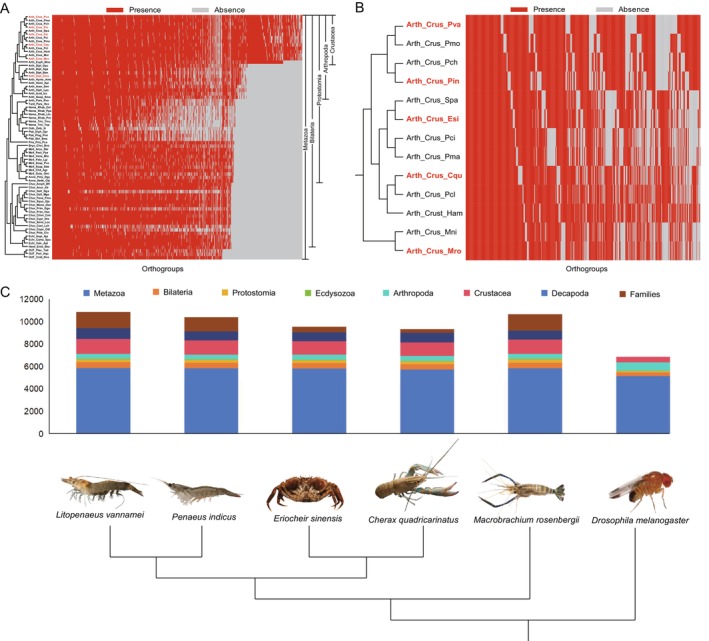
Comparative genomic analysis of Crustacea species. (A) Orthogroup presence and absence across 70 species; (B) Orthogroup presence and absence of 13 crustaceans; (C) Phylostratum‐specific gene in five Crustacea aquatic species.

Through a comprehensive gene homology comparison analysis, a total of 1,563,253 genes from 70 species were classified into 81,557 orthogroups (Figure 1A, Tables S2 and S3). For all analyzed species and genes, the classification revealed that 12,272 orthogroups are derived from Metazoa‐origin genes, 5615 orthogroups are associated with Arthropod‐origin genes, and 5543 orthogroups pertain to Crustacea‐origin genes. The remaining 2093 orthogroups represent lineage‐specific genes, including those from the families Penaeidae, Caridea, Astacidea, Anomura, Branchyura. For the species examined (using the *L. vannamei* as an example), a total of 24,978 genes were divided into 10,878 orthogroups. Among these, 5861 orthogroups are of Metazoa origin, 437 orthogroups are of Arthropod origin, and 1334 orthogroups are of Crustacea origin (Figure 1B). Genes of Decapod origin comprise 955 orthogroups, while the remaining 1448 orthogroups are specific to the family Penaeidae (Table 1 and Figure 1C). We selected 78 orthologs that are present in all 70 species to construct a phylogenetic tree for analyzing the evolutionary relationships among these species. Unsurprisingly, the phylogenetic tree aligns with the established taxonomic relationships of all species (Figure S1).

**TABLE 1:** Phylostratum‐specific genes number of six species.

Phylostage	*L. va*	*P. in*	*E. si*	*C. qu*	*M. ro*	*D. me*
Metazoa	5861	5849	5824	5732	5860	5156
Bilateria	538	504	491	477	502	310
Protostomia	254	253	249	253	267	152
Ecdysozoa	51	39	47	45	59	30
Arthropoda	437	431	446	441	458	739
Crustacea	1334	1257	1185	1197	1273	499
Decapoda	955	786	796	884	791	—
Families	1448	1280	513	320	1448	—

*Note:* *L. va*: *L. vanamei*; *P. in*: *P. indicus* ; *E. si*: *E. sinesis*; *C. qu*: *C. quadricarinatus* ; *M. ro*: *M. rosenbergii* ; *D. me*: *D. melanogaster* .

### 3.2 Comparative Transcriptome Under Cold Stress of Crustacean Species

To explore the regulatory mechanisms of gene expression in crustacean aquatic animals under low‐temperature stress, we analyzed the transcriptomes of five representative species: two Penaeidae shrimp, *L. vannamei* (Pacific white shrimp) and *P. indicus* (Indian white shrimp); one Brachyura, *Eriocheir sinensis* (Chinese mitten crab); one Astacidea, *Cherax quadricarinatus* (Redclaw crayfish); and one Caridea, *Macrobrachium rosenbergii* (Giant freshwater prawn). As the best‐annotated arthropod model species, possessing a highly comprehensive functional annotation system and serving as an appropriate phylogenetic outgroup, *Drosophila melanogaster* was included as a comparative reference in this study. From the homologous gene matrix, we identified 4711 orthogroups that are conserved across the five crustacean species and selected the longest transcript as the representative for comparative transcriptomic analysis. PCA revealed that, despite the inclusion of both low‐temperature stress and non‐stress control samples for each species, inter‐species separation was markedly greater than the within‐species separation between stress and control conditions. This indicates that species identity explains a larger proportion of the variance in global transcriptomic profiles than the cold‐stress treatment under the present experimental design (Figure S2).

Following the differential gene expression analysis, it was observed that the five crustacean species exhibited significant upregulation of 913, 1114, 768, 502, and 1135 genes, respectively, under low‐temperature stress, while 733 to 1295 genes were significantly downregulated (Table 2). Among these species, *M. rosenbergii* displayed the highest number of responsive genes, while *E. sinensis* exhibited the fewest. In *Drosophila*, the number of downregulated genes significantly exceeded that of upregulated genes, with only 299 genes upregulated and 4431 genes downregulated. Analysis at the gene family level revealed that *E. sinensis* had the fewest orthogroups responding to low‐temperature stress, whereas the two shrimp species contained more orthogroups, indicating that *M. rosenbergii* did not possess the largest gene families in response to low‐temperature stress.

**TABLE 2:** Significant up and down regulated gene under low‐temperature across six species.

Species	Gene		Ortho groups	
	Up	Down	Up	Down
*L. vannamei*	913	1034	503	506
*P. indicus*	1114	898	641	441
*C. quadricarinatus*	768	787	347	365
*E. sinensis*	502	733	162	249
*M. rosenbergii*	1135	1295	390	448
*D. melanogaster*	299	4431	46	1280

**FIGURE 2:**
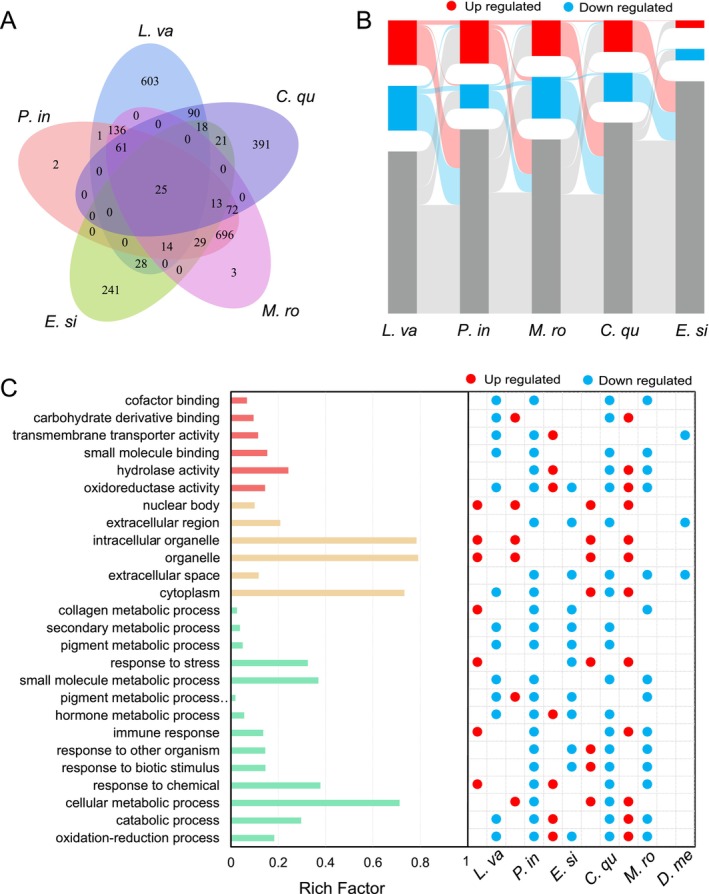
Transcriptome analysis of low temperature stress in Crustacea aquatic species. (A) Venn diagram illustrating the significant differentially expressed genes belonging to orthogroups of five Crustacea aquatic species. (B) Sankey diagram of the significant upregulated and downregulated genes belonging to orthogroups under low‐temperature stress in five Crustacea aquatic species. *C. crustacea* aquatic species shared significant enriched GO term.

Comparative analysis at the orthogroup level showed that there were 25 orthogroups containing significantly different genes under low‐temperature stress across all five crustacean species (Figure 2A), with 61 orthogroups shared by *L. vannamei* , *P. indicus* , *C. quadricarinatus* , and *M. rosenbergii* ; 14 orthogroups shared by *L. vannamei* , *P. indicus* , *E. sinensis* , and *M. rosenbergii* ; and 13 orthogroups shared by *L. vannamei* , *E. sinensis* , *C. quadricarinatus* , and *M. rosenbergii* . The Sankey diagram further indicates that only a small proportion of gene families are significantly differentially expressed among all five crustacean species (Figure 2B). Through gene‐level comparative analysis, no significantly different genes under low‐temperature stress were identified that are common to all five crustacean species; only a few genes were responsive in two or three species.

### 3.3 Functional Characteristics of Genes Responding to Low‐Temperature Stress

Through GO enrichment analysis of upregulated and downregulated genes following low‐temperature stress across six species, we prioritized the presentation of GO terms significantly enriched in at least three species (Figure 2C). These terms primarily include “Oxidation–reduction processes,” “Catabolic processes,” “Responses to chemicals,” “Hormone metabolic processes,” “Collagen metabolic processes,” “Intracellular organelles,” “Hydrolase activities,” and “Carbohydrate derivative binding.” Following KEGG enrichment analysis, we selectively presented KEGG pathways that were significantly enriched in at least three species, including “Pantothenate and CoA biosynthesis,” “Pattern recognition receptors,” “Lysosomes,” “Spliceosomes,” “Steroid hormone biosynthesis,” “Cytochrome P450,” and “Glycolysis/Gluconeogenesis.” The Gene Ontology (GO) functions and Kyoto Encyclopedia of Genes and Genomes (KEGG) pathways identified in this study are significantly enriched across multiple species.

Notably, the analysis demonstrated a higher prevalence of non‐shared GO terms and KEGG pathways (Table 3). For instance, in *L. vannamei* , the “Aminoacyl‐tRNA biosynthesis” and “AMPK signaling pathways” exhibited significant enrichment among upregulated genes, while “Fatty acid biosynthesis” and “Purine metabolism” were notably enriched in downregulated genes. In *P. indicus* , “Zeatin biosynthesis” and “Protein phosphatases and associated proteins”, were significantly enriched among upregulated genes, whereas “Arachidonic acid metabolism” and “Aminobenzoate degradation” were markedly enriched in downregulated genes. In *E. sinensis* , “Antifolate resistance” and “chaperones and folding catalysts” showed significant enrichment in upregulated genes, while “Terpenoid backbone biosynthesis” and the “Biosynthesis of ansamycins” were significantly enriched in downregulated genes. In *C. quadricarinatus* , the “MAPK signaling pathway” and “Ubiquitin‐mediated proteolysis” significantly enriched upregulated genes, while “Oxidative phosphorylation” and the “Biosynthesis of phenylalanine, Tyrosine, and Tryptophan” were significantly enriched in downregulated genes. In *M. rosenbergii* , “Messenger RNA biogenesis” and “Proteasome pathways” significantly enriched upregulated genes, while “Mineral absorption” and “Penicillin and cephalosporin biosynthesis” were associated with downregulated genes. In *D. melanogaster* , “Neuroactive ligand‐receptor interaction” and “Circadian rhythm” were significantly enriched among upregulated genes, while the “Citrate cycle” and “the functioning of Cilium and associated proteins” significantly enriched downregulated genes.

**TABLE 3:** KEGG pathway of low‐temperature stress responded gene across six species.

ID	Pathway	*L. va*	*P. in*	*E. si*	*C. qu*	*M. ro*	*D. me*
map00770	Pantothenate and CoA biosynthesis	**↓**	**↓**	**↓**	**↓**	**↓**	
map04054	Pattern recognition receptors	**↓**	**↓**		**↓**	**↓**	**↓**
map04091	Lectins		**↓**	**↓**	**↓**	**↓**	**↓**
map04142	Lysosome		**↓**		**↑ ↓**	**↓**	**↓**
map03041	Spliceosome	**↑**	**↑**	**↑**		**↑**	
map00310	Lysine degradation	**↓**		**↑**		**↑ ↓**	
map00040	Pentose and glucuronate interconversions	**↓**	**↓**	**↓**	**↓**		
map00120	Primary bile acid biosynthesis	**↓**	**↓**		**↓**	**↓**	
map00140	Steroid hormone biosynthesis	**↓**	**↓**		**↓**	**↓**	
map00199	Cytochrome P450	**↓**	**↓**		**↓**	**↓**	
map00830	Retinol metabolism	**↓**	**↓**		**↓**	**↓**	
map00981	Insect hormone biosynthesis	**↓**	**↓**	**↓**	**↓**		
map02000	Transporters	**↓**	**↓**		**↓**	**↓**	
map00010	Glycolysis/Gluconeogenesis	**↓**	**↓**			**↑**	**↓**
map00330	Arginine and proline metabolism	**↓**	**↓**		**↓**		**↓**
map00620	Pyruvate metabolism	**↓**	**↓**			**↓**	**↓**
map04146	Peroxisome	**↓**	**↓**			**↓**	
map04215	Apoptosis‐multiple species	**↑**			**↓**	**↓**	
map04727	GABAergic synapse	**↓**	**↓**	**↑**			
map00531	Glycosaminoglycan degradation		**↓**		**↓**	**↓**	
map00537	Glycosylphosphatidylinositol (GPI)‐anchored proteins	**↓**	**↓**			**↓**	
map00630	Glyoxylate and dicarboxylate metabolism	**↓**	**↓**	**↑**			
map00052	Galactose metabolism	**↓**	**↓**		**↓**		
map00053	Ascorbate and aldarate metabolism	**↓**			**↓**	**↓**	
map00130	Ubiquinone and other terpenoid‐quinone biosynthesis	**↓**	**↓**		**↓**		
map00220	Arginine biosynthesis	**↓**	**↓**		**↓**		
map00232	Caffeine metabolism	**↓**			**↓**	**↓**	
map00250	Alanine, aspartate, and glutamate metabolism	**↓**	**↓**		**↓**		
map00360	Phenylalanine metabolism	**↓**			**↓**	**↓**	
map00790	Folate biosynthesis	**↓**	**↓**		**↓**		
map01004	Lipid biosynthesis proteins	**↓**			**↓**	**↓**	
map04922	Glucagon signaling pathway	**↓**	**↑**				**↓**
map04976	Bile secretion	**↓**		**↑**			**↓**

*Note:* *L. va*: *L. vanamei*; *P. in*: *P. indicus* ; *E. si*: *E. sinesis*; *C. qu*: *C. quadricarinatus* ; *M. ro*: *M. rosenbergii* ; *D. me*: *D. melanogaster* . Blue arrows indicate pathways enriched with a greater number of downregulated genes, whereas red arrows indicate the opposite.

### 3.4 Low‐Temperature Stress Responses Genes in Crustacean Species

**FIGURE 3:**
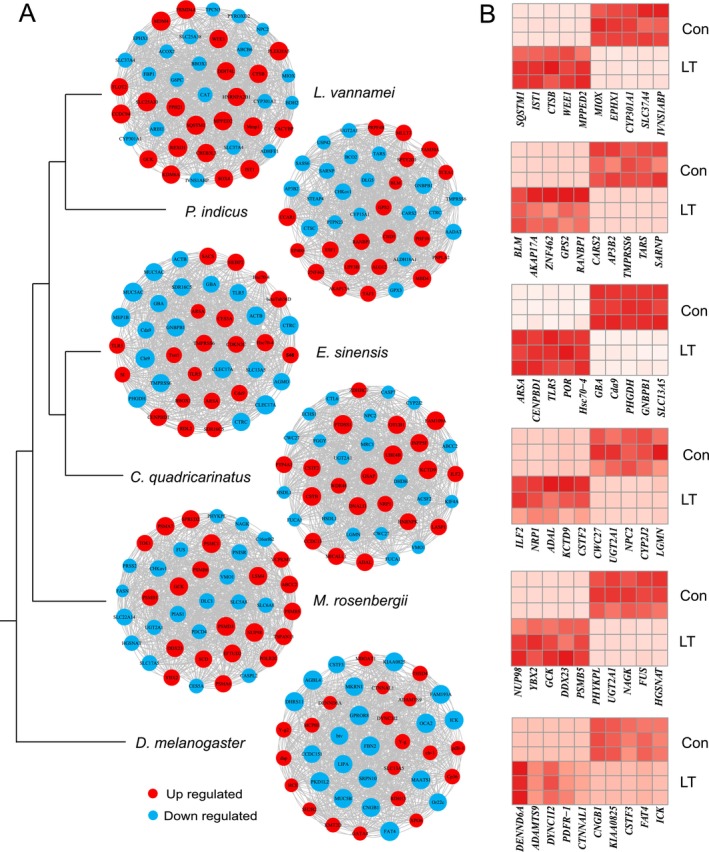
Key gene identification of five Crustacea aquatic species and *D. melanogaster* by WGCNA co‐expression network. (A) Hub genes of low‐ temperature stress significant responded gene module. (B) Expression pattern of top five up and down annotated genes under low‐temperature stress.

By constructing a gene expression regulatory network after stress, transcripts from six species were classified into 10 to 19 modules according to their expression patterns. Using enrichment and hypergeometric tests on differential genes across the modules, we identified significant upregulated and downregulated modules that respond to low‐temperature stress for each species. Based on the connectivity within these modules, we selected 40 key genes with homologous annotations from the co‐expression network of each species (Figure 3A, Table 4). Following the occurrence of low‐temperature stress, the biological response is multifaceted and complex. Previous research has characterized this response process, classifying it into primary, secondary, and tertiary responses based on both response time and type (Donaldson et al. 2008; Ren et al. 2021). And some genes are pleiotropic and may participate in multiple biological processes depending on the species and the stage of the stress response. Therefore, our classifications were based on each gene's dominant functional annotation and its relative position within the response cascade. Through homologous gene annotation and literature review, stress response genes can be categorized (Table 4).

**TABLE 4:** Low‐temperature responded genes across six species.

Biological response		Crustaceans shared	*L. va*	*P. in*	*E. si*	*C. qu*	*M. ro*	*D. me*
Primary response	Neuroendocrine or corticosteroid response	*EcKL*	—	*ZNF462*, *AP3B2*	—	*NPC2*, *CYP2J2*	*EcKL*	—
	Other signaling response	*TMPRSS6*, *FCN2*, *CASP7*, *DHX7*, *RPTPS*, ***PTPRS***	*—*	*GPS2*, *TMPRSS6*	*—*	*NRP1*	*—*	*DENND6A*, *CNGB1*, *FAT4*, *KIAA0825*
Secondary response	Metabolic response	*CYP6A9*, *PRSS2*, *CYP2W1*, *SLC22A14*, *SULT*, *UGT2A1*, *ASTL*, *MCT1*, *SLC5A8*, *ABCA3*, *BDH1*, *SLC25A20*, *BCO2*, *CROT*, *AADAT*, ***CLH***	*CTSB*, *MPPED2*, *MIOX*, *EPHX1*, *SLC37A4*	*RANBP1*, *CARS2*, *TARS*	*ARSA*, *GBA*, *CDA9*, *SLC13A5*	*ADAL*, *UGT2A1*	*GCK*, *PSMB5*, *PHYKPL*, *NAGK*, *HGSNAT*	*ICK*
	Immunological change	*HSPA5*, *TLR5* , ***NLRP10*** , *SAMHD*	*IVNS1ABP*	*—*	*TLR5* , *GNBPB1*	*ILF2*, *LGMN*	*—*	—
	Antioxidant change	*AKR*, *FAO*, *DHRS11*, *SARDH*, *GSTM3*, *CYB5*	*—*	—	—	—	—	—
	Cell cycle response	*SPDEF*, *CENPE*, *CCAR1*	*—*	*SARNP*	*—*	—	—	—
	mRNA Splice	—	—	*AKAP17A*	*—*	*CWC27*	*FUS*	*CSTF3*
Tertiary response	Developmental change	*BRAC*, *CES5A*, *HSC70‐4*, *BCAN*, *INX2*, *GABPA*, *EHF*	*WEE1*	*BLM*	*HSC70‐4*	*KCTD9*	*YBX2*, *DDX23*	*ADAMTS9*, *DYNC1I2*, *PDFR‐1*, *CTNNAL1*
	Disease resistance	—	*SQSTM1*, *IST1*	*—*	*POR*	*—*	*NUP98*	*—*
	Change in growth	*CHT5* , *UNC‐54*, *CYP301A1*, *CDA5*, *CHT2*	*CYP301A1*	*—*	—	—	—	—

*Note:* *L. va*: *L. vanamei*; *P. in*: *P. indicus* ; *E. si*: *E. sinesis*; *C. qu*: *C. quadricarinatus* ; *M. ro*: *M. rosenbergii* ; *D. me*: *D. melanogaster* ; ●: Metazoan; ●: Bilateria; ●: Protostomia; ●: Arthropoda; ●: Crustacea.

Following low‐temperature stress, the significantly responsive hub genes in *L. vannamei* primarily focus on the metabolic response category, which includes *CTSB*, *MPPED2*, *MIOX*, *EPHX1*, and *SLC37A4*. The *IVNS1ABP* gene may be implicated in immunological changes, while *WEE1*, *SQSTM1*, and *IST1* are involved in developmental changes, disease resistance, and growth regulation, respectively. In *P. indicus* , the hub genes *TMPRSS6*, GPS, *AP3B2*, and *ZNF462* likely represent upstream regulatory components of stress signaling, with *TMPRSS6* acting as a membrane‐anchored serine protease potentially modulating TGF‐β/BMP‐like cues via proteolytic processing, *GPS* mediating GPCR‐linked MAPK/JNK cascades, AP3B2 shaping signal input through receptor/cargo trafficking, and *ZNF462* functioning as a downstream zinc‐finger transcriptional regulator coordinating signal‐driven transcriptional reprogramming. *RANBP1*, *CARS2*, and TARS engage in metabolic responses; and *SARNP* and *AKAP17A* may be linked to cell cycle responses and mRNA splicing. The responsive genes in *E. sinensis* under low‐temperature stress, associated with metabolic response, immunological changes, developmental changes, and disease resistance, include *ARSA*, *GBA*, *CDA9* and *SLC13A5*, *TLR5*, GNBpB1, and *HSC70‐4* and *POR*, respectively. The signaling response genes related to low‐temperature stress in *C. quadricarinatus* include *NPC2*, *CYP2J2*, and *NRP1*. Additionally, *ADAL*, *UGT2A1*, *ILF2*, *LGMN*, and *CWC27* are involved in metabolic responses, immunological changes, and mRNA splicing. In *M. rosenbergii* , the genes responsive to low‐temperature stress comprise *EcKL*, which is implicated in neuroendocrine or corticosteroid responses; *GCK*, *PSMB5*, *PHYKPL*, *NAGK*, and *HGSNAT*, which contribute to metabolic responses and *FUS*, *YBX2*, *DDX23* and *NUP98*, which are involved in mRNA splicing, developmental changes, and disease resistance. In contrast to the primarily metabolic response genes found in crustaceans, the responsive genes in fruit flies under low‐temperature stress predominantly focus on signaling responses and developmental changes, including *DENND6A*, *CNGB1*, *FAT4*, *KIAA0825*, *ADAMTS9*, *DYNC1I2*, *PDFR‐1*, and *CTNNAL1* (Figure 3B).

### 3.5 Crustacean Species Shared Responsive Genes Under Low‐Temperature

**FIGURE 4:**
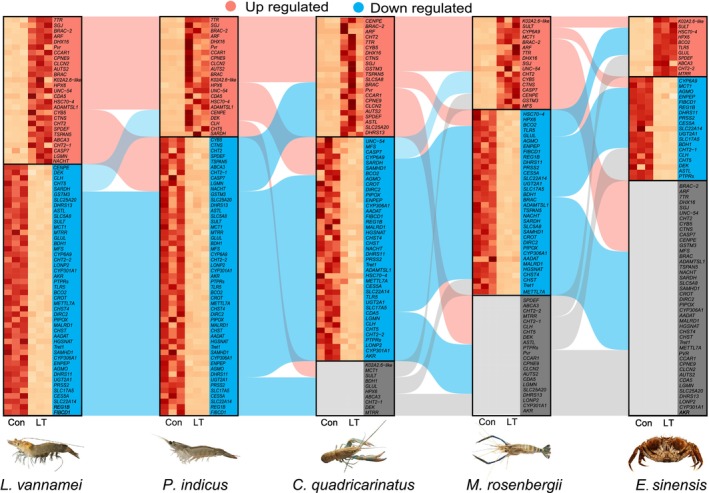
Sankey diagram of significant upregulated and downregulated genes shared among five crustacean aquatic species belonging to Orthogroups under low‐temperature stress.

**FIGURE 5:**
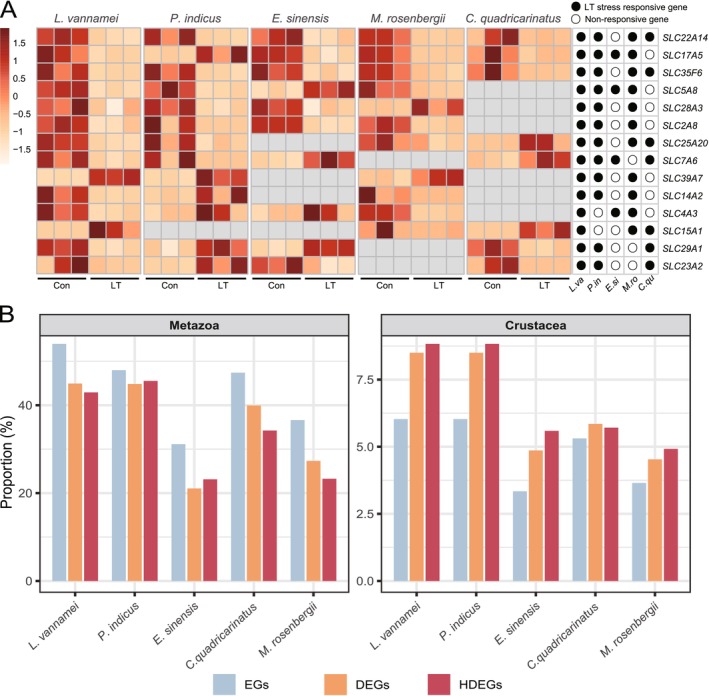
Distribution and expression patterns of genes responsive to low‐temperature stress. (A) SLC genes expression pattern under low temperature stress. (B) Proportion of Metazoa and Crustacea lineage‐specific genes in EGs, DEGs, and HDEGs. DEGs, significantly differential expressed genes; EGs, expressed genes; HDEGs, highly significantly differential expressed genes.

To investigate the shared characteristics of crustaceans in response to low‐temperature stress, we screened for orthogroups common to crustaceans from the homologous gene matrix (where at least four species have responsive genes among five crustacean species, as shown in Table 4, Figures 4 and 5).

Through functional annotation and literature research, we identified a total of 113 orthogroups that contain genes significantly responsive to low‐temperature stress. Genes such as *EcKL*, *TMPRSS6*, *FCN2*, *CASP7*, *DHX7*, *RPTPS*, and *PTPRS* are putatively involved in signaling‐related processes, mapping to ecdysteroid‐linked stress signaling, TGF‐β/BMP–SMAD–like regulation, lectin‐mediated PRR/complement‐like innate signaling, caspase‐dependent apoptotic cascades, spliceosome/RNA‐processing–coupled regulation, and receptor‐type PTP control of MAPK/ERK, JAK/STAT, and insulin/PI3K–AKT axes. The metabolic response category encompasses the greatest number of low‐temperature stress‐responsive genes, including *SLC5A8*, *SLC25A20*, *PRSS2*, *CYP2W1*, *SULT*, *BCO2*, and *CLH*. Genes *HSPA5*, *TLR5*, *NLRP10*, and *SAMHD* are associated with immunological changes; genes such as *AKR*, *FAO*, *DHRS11*, *SARDH*, *GSTM3*, and *CYB5* are included in oxidative changes; the cell cycle response involves *SPDEF*, *CENPE*, and *CCAR1*; moreover, *SLC22A14*, *BRAC*, *CES5A*, *HSC70‐4*, *BCAN*, *INX2*, *GABPA*, *EHF*, *CHT5*, *UNC‐54*, *CYP301A1*, and *CDA5* may play regulatory roles in the development and growth changes of crustaceans. Among these genes, several members of the SLC (solute carrier) gene family have been implicated in the low‐temperature stress response of crustaceans, including *SLC22A14*, *SLC17A5*, *SLC5A8*, and others (Figure 5).

Among the orthogroups that respond to low‐temperature stress in crustaceans, most are classified as “ancient genes” that originated in the genomes of the common ancestors of crustaceans at Metazoa evolutionary nodes, while only a small proportion are considered “young genes” that emerged later at subsequent nodes. For example, the *CCAR1* and *BRAC* genes appeared at the bilateral symmetry animal node, whereas the *CHT2* and *CHT5* genes emerged at the arthropod evolutionary node; the *PTPRS*, *CLH*, and *NLRP10* genes, on the other hand, emerged specifically at the Crustacea evolutionary node. To further characterize differences in the magnitude of differential expression between young and ancient genes, we assessed not only the proportion of differentially expressed genes (DEGs) across gene categories under the standard criteria, but also the proportion of highly differentially expressed genes (HDEGs) under more stringent criteria (Section 2.3). The results showed that, in each species, the proportions of DEGs and HDEGs exhibited a progressively increasing trend from the ancient category (Metazoa) to the younger category (Crustacea) (Figure S3, Table S4). In the oldest and most conserved gene category (Metazoa), the proportions of DEG/HDEG relative to EGs were reduced in nearly all five species, whereas in the “younger” gene category (Crustacea), the proportions of DEGs/HDEGs relative to EGs were consistently increased (Figure 5B).

## 4 Discussion

Temperature is a primary limiting factor in aquatic ecosystems, significantly influencing the foundation of life (Carter et al. 2023). Ectothermic aquatic animals, including fish, mollusks, and crustaceans, depend on their environment to regulate body temperature (Abram et al. 2017). For instance, fish exhibit physiological and molecular adaptations to low‐temperature stress through multidimensional collaborative mechanisms (Wang, Liu, and Ma 2022). Key strategies involve the synthesis and functional optimization of antifreeze proteins (AFPs) (El‐Sayed et al. 2023; Zhifeng et al. 2023). Mollusks also adapt to low‐temperature stress by regulating energy metabolism, antioxidant defense mechanisms, damage repair systems, and heat shock proteins (Lin et al. 2025; Nie et al. 2023; Zhang et al. 2024).

Within the temperature tolerance range of crustaceans, warmer temperatures can accelerate growth and maturation, while colder temperatures can slow or even halt these processes. Previous research on low‐temperature stress in crustaceans, particularly represented by *L. vannamei* , has demonstrated that cold conditions regulate amino acid metabolism, lipid metabolism, antioxidant activity and immune system functions (Fan et al. 2013; Qiu et al. 2011; Zhu et al. 2024). However, these studies have primarily focused on data from individual species, resulting in the identification of low‐temperature regulatory genes that display significant variations exclusively within specific species. Given that animals within the same crustacean group often share similar biological characteristics, conducting a synchronous analysis across multiple species to identify shared response genes is crucial for advancing research on low‐temperature stress in crustaceans. Therefore, this study analyzed transcriptomic data related to low‐temperature stress from multiple representative crustacean species while performing comparative genomic analysis to investigate both the shared and specific key genes involved in the low‐temperature stress response. A comprehensive comparative genomic analysis involving multiple species is essential for deciphering shared low‐temperature stress response genes in crustaceans. By taking into account both the evolutionary representativeness of the selected species and the availability of published, publicly accessible genome data suitable for robust analysis, we compiled complete gene sets from the genomes of 70 eukaryotic species. This comprehensive dataset enabled the identification of more than 80,000 orthogroups derived from all gene clusters. These orthogroups provide the foundational data necessary for conducting comparative transcriptomic analyses.

The GO and KEGG analyses revealed consistent functional enrichment across species, mainly involving oxidation–reduction, hormone metabolism, and spliceosome‐related processes, supporting the idea that energy metabolism and transcriptional regulation are central to cold‐stress adaptation. Nevertheless, only 25 orthogroups contain significantly different genes present in all five crustaceans. Furthermore, no shared significantly different homologous genes were identified, indicating that the regulatory gene networks responding to low‐temperature stress differ markedly among species. This disparity may be attributed to the complexity of biological processes that change under stress conditions and the large number of genes involved. To explore shared genes across crustaceans, we identified 113 orthogroups that are common to at least four of the five Crustacea species. The five focal taxa span multiple decapod lineages (Penaeidae, Caridea, Brachyura, Astacidea) and contrasting habitat/osmoregulatory strategies relevant to cold tolerance, ranging from marine/brackish penaeid shrimps ( *L. vannamei* , *P. indicus* ) to euryhaline catadromous *E. sinensis* and predominantly freshwater *C. quadricarinatus* and *M. rosenbergii* . Across species, we observed convergent GO/KEGG enrichments but little overlap of DEGs at the gene/orthogroup level, indicating pathway‐level conservation accompanied by species‐specific recruitment of molecular nodes. These patterns suggest that cold‐stress regulation is jointly shaped by phylogenetic history and eco‐physiological background.

To gain a deeper understanding of the functional characteristics of these low‐temperature stress response genes, we identified their functions through homology annotation and a review of relevant literature. We then consulted previous studies focused on the effects of low temperature on the physiological responses of other ectothermic aquatic animals. The genes were classified into three categories: Primary Response (Neuroendocrine, Corticosteroid, and other signaling responses), Secondary Response (Metabolic, Immunological, Antioxidant changes, Cell cycle responses, mRNA splicing), and Tertiary Response (Development, Disease, and Growth changes) (Donaldson et al. 2008; Ren et al. 2021).

Following low‐temperature stress, animals transmit regulatory signals through multiple signaling pathways, resulting in complex cascade responses. The genes involved include *EcKL*, *RPTPS*, and *PTPRS*. *EcKL* regulated oxidative stress through pathways mediated by ecdysteroid phosphorylation (Scanlan et al. 2022). *RPTPS* induces insulin signaling cascades via the expression of protein tyrosine phosphatases (Sevillano et al. 2021). *PTPRS* modulates pathways such as *STAT3* and *ERK* to trigger cellular proliferation and apoptosis (Kim et al. 2018; Li et al. 2016).

Thermal stress induces metabolic reprogramming, and the genes identified in this study that are associated with metabolic responses play significant roles in crustaceans (Wang, Zhang, et al. 2022). For example, multiple members of the *CYP* gene family are associated with hormone metabolism and toxin degradation under stress conditions in *Agasicles hygrophila* and *Liriomyza trifolii* (Wang et al. 2021; Wang et al. 2024). *UGT* genes are involved in metabolic activities related to temperature stress in *Palaemon graviera* and *Salmo salar* (Nuez‐Ortín et al. 2018; Shi et al. 2022). Consistent with previous studies, this research also identified genes related to immunological and antioxidant changes, such as the families of *HSP* (Jahan et al. 2023; Ji et al. 2016) and *TLR* (Sousa et al. 2022; Wojda 2017), which have been found to participate in low‐temperature stress through immune regulation across multiple taxa.

In Tertiary response, the biological response reflected in changes in development and growth. Molting (Ecdysis) is the defining character of Ecdysoza (Arthropods, Nematodes and related phyla). *CHT*, *CYP301*, *CDA* genes differential expressed imply the important role in peculiar growth changes under low temperature. The *BRAC* genes are involved in DNA damage repair during individual development (Varol et al. 2018), while *CES* genes participate in the development of male reproductive organs and sperm formation (Ru et al. 2015).

Our analysis revealed that gene evolutionary age was associated with the degree of transcriptional responsiveness to cold stress. Across all five species, both DEGs and HDEGs proportions showed a progressive increase from ancient gene classes to younger lineage‐specific classes. This indicates that younger genes tend to display greater transcriptional plasticity than ancient conserved genes. One possible explanation is that ancient genes, which are often involved in core cellular and developmental functions, are under stronger purifying selection and therefore maintain more stable expression patterns. In contrast, younger genes may be more lineage‐specific and functionally flexible, making them more readily recruited into species‐specific stress responses. These findings suggest that evolutionary novelty contributes to cold‐stress transcriptional divergence, even though convergent responses can still be observed at the pathway or module level (Popadin et al. 2014; Schlötterer 2015; Zhao et al. 2024).

The SLC family is recognized as a key regulator of metabolism and is closely linked to osmoregulatory processes. By transporting small molecules and ions, SLCs play a critical role in maintaining cellular metabolism and homeostasis (Khan et al. 2025; Lin et al. 2015). The SLC family consists of 450 proteins classified into 70 families and can also be specifically categorized according to their substrate functions. Previous studies have indicated that the SLC gene family exhibits differential expression in response to pathogenic stimuli and salinity stress in crustaceans, investigations on SLC‐mediated temperature adaptive regulation remain relatively limited (Ge et al. 2019). In this study, we discovered that SLCs exhibit specific expression patterns under low‐temperature stress. Based on functional research conducted in mammals, we hypothesize that SLCs may also play an important role in temperature adaptive regulation in crustaceans. Under low‐temperature stress conditions, five SLC members (*SLC5A8*, *SLC22A14*, *SLC25A20*, *SLC35F6*, *SLC7A6*) displayed responses to low temperature stress across four species in five species. *SLC5A8* is primarily involved in short‐chain fatty acid metabolism, intestinal nutrient absorption, and metabolic processes; *SLC22A14* mediates Zn^2+^ homeostasis and spermatogenesis; and *SLC25A20* participates in fatty acid oxidation that regulates energy supply (Gurav et al. 2015; Kuang et al. 2021; Yuan et al. 2021). Current research on the SLC gene family in crustaceans remains at a preliminary stage, our analytical results further demonstrate its significant regulatory potential in responding to environmental stressors. The current study revealed differential expression of all 14 investigated SLC genes in *L. vannamei* , a species demonstrating remarkable salinity adaptability and temperature regulation capacity. Meanwhile, we listed the specific SLC family members involved and their putative functions in low‐temperature homeostatic regulation in Table S5. This finding aligns with previous research, suggesting a potential functional linkage between thermoregulation and osmoregulation mechanisms in crustaceans (Jaffer et al. 2024). Such efforts will substantially advance our understanding of the adaptive mechanisms governing temperature and salinity tolerance in crustaceans.

Among the low‐temperature‐related genes, we also identified several phylostratum‐specific genes across different taxa, which may be associated with distinct biological processes. The gene we discovered may contribute uniquely to processes in crustaceans (de Oliveira et al. 2019). For instance, *NLRP10* can form inflammasomes that respond to the immune system (Chang‐Xi et al. 2024; Li et al. 2022; Sun et al. 2024). Chitinase (*CHT*) has been linked to the formation of the cuticular exoskeleton and the intestinal peritrophic matrix, which are unique structures in Arthropoda (Zhang et al. 2021; Zhou et al. 2018). Additionally, the chlorophyllase (*CLH*) genes, originating from plants, have been identified in all five Crustacea species examined in this study and are significantly differentially expressed in response to low‐temperature stress. These genes may have been integrated into the ancestral genomes of crustaceans through horizontal gene transfer prior to their evolutionary divergence (Boto 2014; Wybouw et al. 2016).

The temperature adaptation range is a critical agricultural trait in aquaculture varieties. Consequently, enhancing tolerance to low‐temperature stress has emerged as a significant challenge in aquaculture breeding. This study utilizes comparative transcriptome analysis of Crustacea species to identify a range of regulatory genes that respond to low‐temperature stress. Examining the expression and functions of these genes and their corresponding proteins, which are affected by temperature fluctuations, is crucial for elucidating the mechanisms underlying temperature adaptability in crustaceans and for the development of new low‐temperature‐tolerant varieties. Future research that combines gene family analysis, identification, and molecular biology experiments will be essential to validate the candidate key genes identified in this study, thereby enhancing our understanding of the adaptive mechanisms of crustaceans to temperature and salinity.

## 5 Conclusion

This study offers significant insights into the gene regulatory mechanisms of crustaceans in response to low‐temperature stress, a critical factor influencing aquaculture yields. Through a comparative transcriptomic analysis of five economically important species, we identified a variety of genes associated with low‐temperature stress responses and their corresponding pathways. Our findings demonstrate both shared and species‐specific gene responses, underscoring the complexity of regulatory networks within crustaceans. The identification of key genes involved in metabolic, immunological, and developmental processes under cold stress enhances our understanding of temperature adaptability. This research establishes a foundation for breeding low‐temperature‐tolerant strains, ultimately contributing to the sustainability of the aquaculture industry.

## Funding

This work was supported by National Natural Science Foundation of China (32402997), Sanya Yazhou Bay Science and Technology City (SKJC‐2022‐PTDX‐025), Hainan Seed Industry Laboratory (B24H10036, B24YQ0009 and B24H10033), and Key R&D Project of Hainan Province (ZDYF2023XDNY176).

## Ethics Statement

The animal study protocol was approved by the College of Marine Life Sciences, Ocean University of China Institutional Animal Care and Use Committee on October 10, 2018 (Project Identification Code: 20181010).

## Conflicts of Interest

The authors declare no conflicts of interest.

## Supporting information


**Figure S1:** Phylogenetic tree for 70 species based on 78 coding orthologs.

**Figure S2:** PCA plot of the transcriptome based on 4711 homologous genes from five Crustacea aquatic species and *D. melanogaster*.

**Figure S3:** Proportional increase in a given gene category among DEGs and HDEGs relative to all expressed genes. DEGs, significantly differential expressed genes; HDEGs, highly significantly differential expressed genes.

**Table S1:** Taxonomic classification, gene numbers, transcriptome data sources, and experimental conditions of the species used in this study.

**Table S2:** Gene numbers in each species for each orthogroup.

**Table S3:** Orthogroup classification of genes in each species.

**Table S4:** Gene number and proportion in a given gene category among EGs, DEGs, and HDEGs.

**Table S5:** Functional characteristics of 16 SLC genes.

## Data Availability

The gene annotations, orthogroups (Tables S2 and S3), and TPM matrixes utilized in this study have been deposited in FigShare, accessible at the following link: https://doi.org/10.6084/m9.figshare.28928312.v2.
