# Supplementary figures and images for: Investigating Low‐Temperature Stress Responses in Crustacea Aquatic Species Through Comparative Transcriptomics
Source: Evol Appl. 2026 May 12;19(5):e70254. doi: 10.1111/eva.70254 (PMC13163145; doi:10.1111/eva.70254)

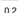

Supplement: Supplementary file 1 — Figure S1: Phylogenetic tree for 70 species based on 78 coding orthologs. [file EVA-19-e70254-s003.pdf]

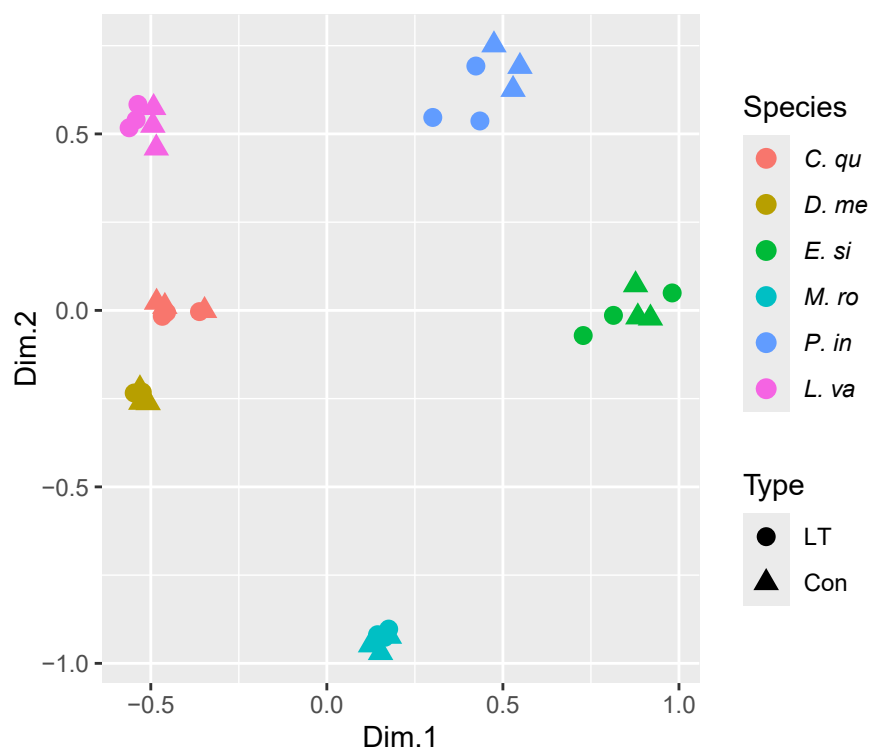

Supplement: Supplementary file 2 — Figure S2: PCA plot of the transcriptome based on 4711 homologous genes from five Crustacea aquatic species and D. melanogaster. [file EVA-19-e70254-s001.pdf]

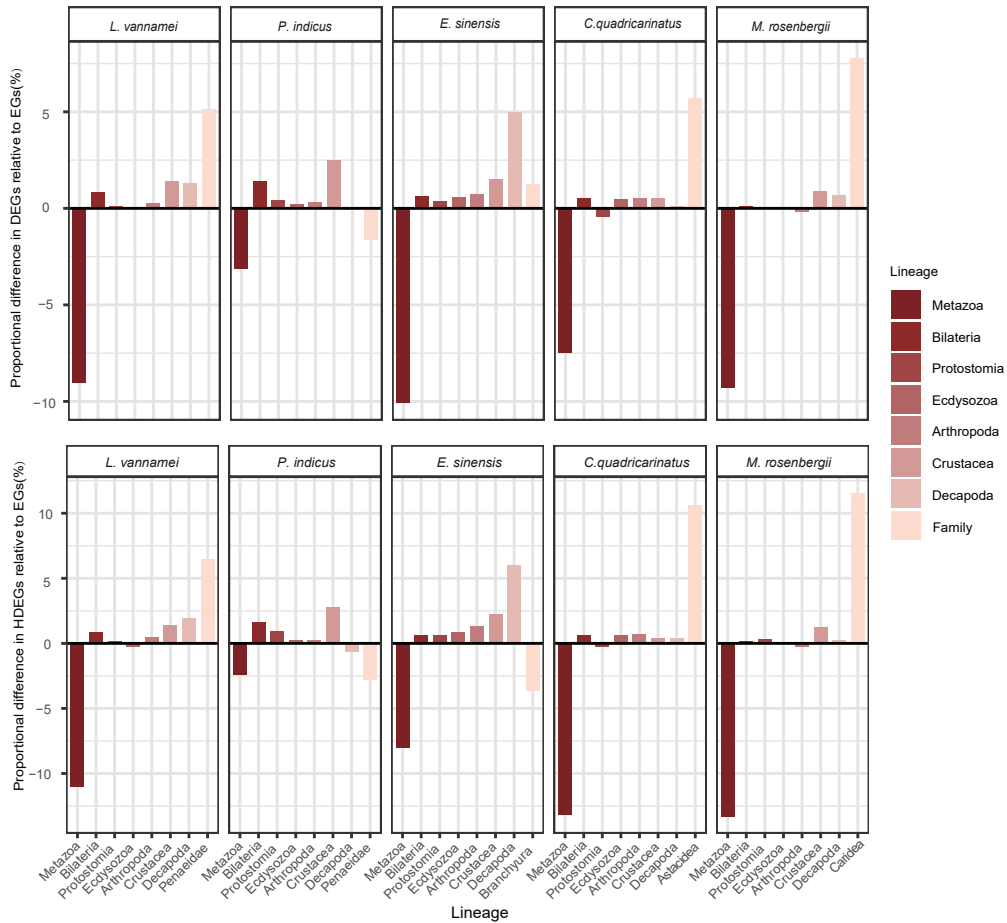

Supplement: Supplementary file 3 — Figure S3: Proportional increase in a given gene category among DEGs and HDEGs relative to all expressed genes. DEGs, significantly differential expressed genes; HDEGs, highly significantly differential expressed genes. [file EVA-19-e70254-s005.pdf]
